# Supplementary material for: A Longitudinal, Population-Level, Big-Data Study of Helicobacter pylori-Related Disease across Western Australia
Source: J Clin Med. 2019 Nov 1;8(11):1821. doi: 10.3390/jcm8111821 (PMC6912511; doi:10.3390/jcm8111821)
Supplement: Supplementary file 1 [file jcm-08-01821-s001.pdf]

Supplementary Data for "*Helicobacter pylori* hotspots: Epidemiology of *Helicobacter pylori* related disease in Western Australia"

Data Sets Obtained from the Australian Bureau of Statistics

- 2011 Australian Census (data for state of Western Australia)
  - 2011Census_B01_WA_SA2_short.csv
  - 2011Census_B07_WA_SA2_short.csv
  - 2011Census_B16A_WA_SA2_short.csv
  - 2011Census_B16B_WA_SA2_short.csv
  - 2011Census_B37_WA_SA2_short.csv
  - POA_2011_AUST.csv
- 2010 and 2015 Population Estimates for state of Western Australia
  - 32350ds0005_wa_2010_2015.csv

Table 1: Age distribution of UBT positive individuals. Based on a binomial distribution statistic, a negative p-value indicates a count of UBT-positive people below the expected number, while a positive p-value is a count above the expected number. Age ranges follow those used in 2011 Census tables, except the 0-4 age range, which has been ignored due to small numbers

Age Range	N UBT_pos	UBT_pos%	WApop%	Signed p-value
(5 .. 14)	620	0.0390	0.1293	-0
(15 .. 19)	488	0.0307	0.0663	-1.483e-89
(20 .. 24)	866	0.0545	0.0710	-2.071e-17
(25 .. 34)	2730	0.1717	0.1445	7.362e-22
(35 .. 44)	3098	0.1948	0.1465	8.581e-62
(45 .. 54)	3040	0.1912	0.1385	1.246e-75
(55 .. 64)	2479	0.1559	0.1134	9.091e-59
(65 .. 74)	1607	0.1011	0.0681	1.879e-54
(75 .. 84)	782	0.0492	0.0394	5.051e-10
(85 ..)	171	0.0108	0.0153	-4.557e-07

Table 2: Distribution of negative, positive and borderline UBT test result counts by age range, together with the ratio of borderline to determinate (i.e positive plus negative) results

Age Range	N UBT_neg	N UBT_border	N UBT_pos	Borderline Ratio
5 - 14	1877	768	528	0.1996
15 - 19	2762	575	316	0.0947
20 - 24	3944	1037	351	0.0705
25 - 34	10860	3330	898	0.0633
35 - 44	12010	3942	1019	0.0639
45 - 54	12789	3859	1164	0.0699
55 - 64	11867	3258	1347	0.0891
65 - 74	7984	2108	1117	0.1107
75 - 84	3306	1020	660	0.1526
85 -	581	209	160	0.2025

Table 3: Age distribution of median negative, positive and borderline UBT test values

Age Range	med UBT_neg	med UBT_border	med UBT_pos
5 - 14	15.0	98.0	1189.5
15 - 19	10.0	91.5	1898.0
20 - 24	10.0	84.0	2020.0
25 - 34	8.0	91.0	1998.0
35 - 44	9.0	87.0	2048.5
45 - 54	9.0	90.0	1896.0
55 - 64	10.0	90.0	1736.5
65 - 74	11.0	92.0	1440.5
75 - 84	12.0	92.0	1190.0
85 -	15.0	96.0	833.0

Table 4: List of SA2 Statistical Areas with at least 500 residents in descending count of UBT persons

SA2	SA2 Name	UBT Persons
51086	Dianella	424.054
51045	Bayswater - Embleton - Bedford	303.298
51124	Bentley - Wilson - St James	263.914
51056	Ballajura	237.000
51132	Beckenham - Kenwick - Langford	229.050
51136	Maddington - Orange Grove - Martin	214.201
51138	Forrestfield - Wattle Grove	213.276
51059	Ellenbrook	212.061
51133	Canning Vale - East	208.257
51041	Perth City	207.000
51057	Beechboro	206.410
51137	Thornlie	198.000
51085	Balga - Mirrabooka	180.389
51131	Willetton	176.595
51098	Alexander Heights - Koondoola	172.472
51099	Butler - Merriwa - Ridgewood	171.443
51108	Wanneroo	167.757
51104	Marangaroo	156.520
51105	Mindarie - Quinns Rocks - Jindalee	156.115
51128	Parkwood - Ferndale - Lynwood	154.728
51185	Baldivis	153.000
51047	Morley	151.068
51090	Nollamara - Westminster	150.557
51122	Rivervale - Kewdale - Cloverdale	146.195

51205	Broome	141.434
51097	Yokine - Coolbinia - Menora	139.821
51044	Bassendean - Eden Hill - Ashfield	137.877
51159	South Lake - Cockburn Central	137.672
51103	Madeley - Darch - Landsdale	137.621
51199	Kalgoorlie	127.475
51102	Girrawheen	124.008
51134	Gosnells	122.184
51182	Murdoch - Kardinya	122.145
51012	Eaton - Pelican Point	121.339
51191	Singleton - Golden Bay - Secret Harbour	118.000
51110	Armadale - Wungong - Brookdale	117.025
51166	Fremantle - South	114.321
51188	Rockingham	112.513
51084	Balcatta - Hamersley	109.211
51006	Bunbury	107.532
51093	Stirling - Osborne Park	106.789
51119	East Victoria Park - Carlisle	106.000
51125	Canning Vale - West	103.148
51127	Cannington - Queens Park	100.820
51165	Fremantle	100.721
51181	Melville	100.402
51140	Kalamunda - Maida Vale - Gooseberry Hill	95.948
51210	Geraldton	95.695
51148	Banjup	95.690
51042	Subiaco - Shenton Park	92.615
51135	Huntingdale - Southern River	92.615
51118	Belmont - Ascot - Redcliffe	92.000
51123	Victoria Park - Lathlain - Burswood	92.000
51141	Lesmurdie - Bickley - Carmel	91.648
51107	Tapping - Ashby - Sinagra	91.622
51062	Lockridge - Kiara	91.115
51046	Maylands	91.000
51208	Kununurra	90.771
51206	Derby - West Kimberley	89.000
51089	Karrinyup - Gwelup - Carine	88.759
51100	Carramar	87.000
51225	Albany	86.590
51147	South Perth - Kensington	85.000
51024	Halls Head - Erskine	84.336
51178	Booragoon	83.301
51200	Kalgoorlie - North	83.246
51213	Geraldton - South	78.022
51109	Yanchep	78.000

51014	Harvey	77.867
51169	Bertram - Wellard (West)	77.856
51039	Mount Lawley - Inglewood	77.824
51149	Beeliar	77.510
51095	Tuart Hill - Joondanna	76.874
51027	Mandurah - North	76.418
51187	Port Kennedy	76.000
51020	Pemberton	75.491
51145	Como	74.769
51161	Success - Hammond Park	73.750
51066	Midland - Guildford	73.633
51170	Calista	73.282
51096	Wembley Downs - Churchlands - Woodlands	73.230
51043	Wembley - West Leederville - Glendalough	73.152
51035	Nedlands - Dalkeith - Crawley	73.000
51003	Busselton Region	72.407
51068	The Vines	71.684
51002	Busselton	71.593
51101	Clarkson	71.442
51054	Swan View - Greenmount - Midvale	71.426
51079	Mullaloo - Kallaroo	71.076
51180	Leeming	70.247
51192	Waikiki	70.176
51243	Northam	69.000
51013	Gelorup - Dalyellup - Stratham	68.952
51071	Currambine - Kinross	68.653
51129	Riverton - Shelley - Rossmoyne	68.018
51174	Parmelia - Orelia	66.663
51117	Seville Grove	66.413
51211	Geraldton - East	65.912
51190	Safety Bay - Shoalwater	65.813
51186	Cooloongup	65.487
51193	Warnbro	65.011
51177	Bicton - Palmyra	65.000
51219	East Pilbara	64.275
51220	Karratha	64.028
51070	Craigie - Beldon	61.944
51146	Manning - Waterford	61.231
51040	North Perth	61.000
51072	Duncraig	61.000
51009	Collie	61.000
51048	Noranda	60.116
51217	Northampton - Mullewa - Greenough	60.104
51233	McKail - Willyung	59.752

51143	Mundijong	58.259
51005	Australind - Leschenault	58.133
51019	Manjimup	56.509
51154	Hamilton Hill	56.467
51197	Exmouth	55.890
51212	Geraldton - North	55.291
51207	Halls Creek	54.229
51198	Boulder	54.000
51073	Greenwood - Warwick	54.000
51113	Forrestdale - Harrisdale - Piara Waters	53.746
51115	Mount Nasura - Mount Richon - Bedfordale	52.816
51160	Spearwood	52.118
51049	Chidlow	51.000
51078	Kingsley	50.702
51196	Carnarvon	50.085
51077	Joondalup - Edgewater	49.867
51017	Bridgetown - Boyup Brook	49.000
51023	Greenfields	48.895
51163	Yangebup	48.010
51179	Bull Creek	47.753
51034	Mosman Park - Peppermint Grove	47.445
51152	Coogee	47.102
51239	Gingin - Dandaragan	46.000
51053	Mundaring	46.000
51028	Mandurah - South	45.619
51153	Coolbellup	44.371
51088	Innaloo - Doubleview	44.276
51224	South Hedland	44.012
51223	Roebourne	43.972
51015	Koombana	43.324
51114	Kelmscott	42.336
51025	Mandurah	42.160
51175	Applecross - Ardross	42.077
51092	Scarborough	41.427
51008	College Grove - Carey Park	40.980
51067	Stratton - Jane Brook	39.954
51142	Byford	39.478
51029	Pinjarra	38.182
51238	Dowerin	38.000
51074	Heathridge - Connolly	37.850
51022	Falcon - Wannanup	36.573
51083	Woodvale	35.298
51164	East Fremantle	33.000
51234	Plantagenet	33.000

51036	Swanbourne - Mount Claremont	32.604
51139	High Wycombe	32.246
51031	Claremont (WA)	31.396
51030	City Beach	31.000
51065	Middle Swan - Herne Hill	30.824
51076	Iluka - Burns Beach	30.347
51075	Hillarys	30.173
51222	Port Hedland	29.671
51080	Ocean Reef	29.455
51116	Roleystone	28.620
51051	Helena Valley - Koongamia	27.704
51184	Winthrop	27.002
51228	Denmark	27.000
51227	Bayonet Head - Lower King	26.964
51245	York - Beverley	25.000
51038	Mount Hawthorn - Leederville	24.534
51195	Esperance Region	24.218
51081	Padbury	23.635
51209	Roebuck	23.566
51236	Chittering	23.151
51050	Glen Forrest - Darlington	23.000
51218	Ashburton (WA)	23.000
51094	Trigg - North Beach - Watermans Bay	22.329
51112	Camillo - Champion Lakes	22.201
51230	Katanning	22.000
51018	Donnybrook - Balingup	22.000
51026	Mandurah - East	21.818
51244	Toodyay	21.341
51183	Willagee	21.219
51215	Meekatharra	21.000
51232	Little Grove - Elleker	20.723
51214	Irwin	20.000
51226	Albany Region	19.970
51194	Esperance	19.782
51171	Casuarina - Wellard (East)	19.230
51016	Waroona	19.000
51010	Dardanup	17.000
51082	Sorrento - Marmion	16.980
51156	Jandakot	16.967
51176	Bateman	16.269
51240	Merredin	16.000
51203	Leinster - Leonora	16.000
51007	Capel	14.873
51033	Floreat	14.700

51144	Serpentine - Jarrahdale	14.263
51216	Morawa	14.000
51001	Augusta	13.659
51248	Murray	13.000
51058	Bullsbrook	12.849
51247	Kulin	12.413
51250	Wagin	12.000
51241	Moora	11.000
51032	Cottesloe	10.555
51202	Kambalda - Coolgardie - Norseman	10.279
51021	Dawesville - Bouvard	10.000
51231	Kojonup	10.000
51004	Margaret River	9.341
51221	Newman	7.042
51242	Mukinbudin	7.000
51237	Cunderdin	7.000
51246	Brookton	7.000
51168	Anketell - Wandi	6.968
51061	Hazelmere - South Guildford	6.311
51249	Narrogin	5.587
51229	Gnowangerup	4.000
51060	Gidgegannup	3.659
51162	Wattleup	3.568
51158	North Coogee	3.318
